# The Association of *PLAUR* Genotype and Soluble suPAR Serum Level with COVID-19-Related Lung Damage Severity

**DOI:** 10.3390/ijms232416210

**Published:** 2022-12-19

**Authors:** Ludmila A. Nekrasova, Anna A. Shmakova, Larisa M. Samokhodskaya, Karina I. Kirillova, Simona S. Stoyanova, Elena A. Mershina, Galina B. Nazarova, Kseniya A. Rubina, Ekaterina V. Semina, Armais A. Kamalov

**Affiliations:** 1 Medical Scientific and Educational Centre, Lomonosov Moscow State University, 119192 Moscow, Russia; 2 Koltzov Institute of Developmental Biology, 117334 Moscow, Russia; 3 Faculty of Medicine, Lomonosov Moscow State University, 119192 Moscow, Russia; 4 Institute of Experimental Cardiology, National Medical Research Centre of Cardiology Named after Academician E.I. Chazov, 121552 Moscow, Russia

**Keywords:** *PLAUR*, rs2302524, COVID-19, soluble uPAR, candidate polymorphism study

## Abstract

Uncovering the risk factors for acute respiratory disease coronavirus 2019 (COVID-19) severity may help to provide a valuable tool for early patient stratification and proper treatment implementation, improving the patient outcome and lowering the burden on the healthcare system. Here we report the results of a single-center retrospective cohort study on 151 severe acute respiratory syndrome coronavirus-2 (SARS-CoV-2)-infected symptomatic hospitalized adult patients. We assessed the association of several blood test measurements, soluble urokinase receptor (uPAR) serum level and specific single nucleotide polymorphisms of *ACE* (I/D), *NOS3* (rs2070744, rs1799983), *SERPINE1* (rs1799768), *PLAU* (rs2227564) and *PLAUR* (rs344781, rs2302524) genes, with the disease severity classified by the percentage of lung involvement on computerized tomography scans. Our findings reveal that the T/C genotype of *PLAUR* rs2302524 was independently associated with a less severe lung damage (odds ratio 0.258 [0.071–0.811]). Along with high C-reactive protein, fibrinogen and soluble uPAR serum levels turned out to be independently associated with more severe lung damage in COVID-19 patients. The identified factors may be further employed as predictors of a possibly severe COVID-19 clinical course.

## 1. Introduction

Severe acute respiratory syndrome coronavirus-2 (SARS-CoV-2) is a causative agent of acute respiratory disease coronavirus 2019 (COVID-19), which first appeared in December 2019 in Wuhan, China, and initiated an alarming pandemic worldwide [1]. The clinical manifestations of COVID-19 are remarkably diverse: from asymptomatic or mild infection to severe pneumonia with a ‘‘cytokine storm” and multiple organ failure, requiring admission to the intensive care unit due to a high mortality risk [2,3,4]. However, the biological underpinnings for such a variability remain largely obscure. The pertinent literature has pointed out several predictors for COVID-19-related mortality [5,6,7,8,9,10], yet a large between-patient variability still remains unexplained and may be dependent on inter-individual genetic variations. A global vaccination campaign has played a positive role in the dramatic decrease of COVID-19-related mortality [11]; however, studies revealing the predictors of COVID-19 disease severity are still in demand.

The underlying mechanism behind the onset of COVID-19 infection is the SARS-CoV-2 spike protein (S-protein), binding the host receptor angiotensin-converting enzyme 2 (ACE2). It allows for viral entry into cells [12]. To fuse with the host cell membrane, SARS-CoV-2 requires proteolytic cleavage of the S-protein by serine proteases, primarily the transmembrane protease TMPRSS2 or plasmin [12,13,14,15]. Plasmin-guided proteolysis is also essential for fibrin blood clot degradation and clearance [16]; coagulation disorders are common in COVID-19 and are associated with the disease severity [17]. The plasminogen activator system, known for its substantial role in the pathogenesis of lung injury, comprises tissue plasminogen activator (tPA), urokinase (uPA), its receptor uPAR and plasminogen activator inhibitors (e.g., PAI-1, encoded by *SERPINE1* gene) [18,19,20]. uPA (encoded by the *PLAU* gene) is a serine protease that cleaves inactive plasminogen, converting it into plasmin; on the cell surface, uPA interacts with uPAR (encoded by the *PLAUR* gene), which accelerates the protease catalytic activation [21,22,23,24]. As opposed to tPA, which is mainly involved in intravascular fibrinolysis, uPA tightly controls extravascular coagulation cascade in lungs and substantially contributes to lung injury [20]. uPAR can be cleaved by uPA into its soluble circulating form (suPAR); suPAR presence in serum was found to correlate with various health conditions, including cancer, cardiovascular diseases, diabetes, etc. [25]. The plasminogen activator system is among the candidate targets involved in COVID-19 pathogenesis. Hence, mapping the genetic variants of these proteins can allow for identifying the disease risk factors associated with lung damage severity in COVID-19.

Angiotensin-converting enzyme (ACE), a central component of the renin-angiotensin system, and its insertion/deletion (I/D) gene polymorphism have been linked to hypertension [26], increased cardiovascular risk [27,28], obesity [29] and diabetes [30], all comorbidities strongly associated with adverse COVID-19 outcome. A dominant player in all these comorbidities and COVID-19-induced complications is endothelial dysfunction [31]. In this regard, special attention should be paid to endothelial nitric oxide synthase (eNOS, encoded by the *NOS3* gene), a vasoprotective enzyme crucial for endothelial homeostasis, since its dysfunction is coupled with many vascular diseases [32,33]. eNOS constitutively generates NO, an important antiviral defense component [34,35]; moreover, NO metabolism is closely related to the development of acute respiratory distress syndrome in COVID-19 patients [36].

It is plausible that differences in the expression or activity of the aforementioned proteins, influenced by the presence of polymorphic genetic variants, can account for the disease severity. Here we conducted a population-based candidate polymorphism association study and genotyped *ACE* (I/D), *NOS3* (rs2070744, rs1799983), *SERPINE1* (rs1799768)*, PLAU* (rs2227564) and *PLAUR* (rs344781, rs2302524) polymorphisms in hospitalized patients with confirmed COVID-19, evaluating their association with the percentage of lung involvement on computerized tomography (CT) scans. We identified *PLAUR* rs2302524 as a single nucleotide polymorphism (SNP) associated with lung involvement in COVID-19 patients. The T/C genotype of *PLAUR* rs2302524 was independently associated with less severe lung damage (odds ratio (OR) 0.258 [0.071–0.811]). Elevated routine blood test parameters (C-reactive protein (CRP), fibrinogen) and suPAR serum level were independently associated with more severe lung damage in COVID-19 patients. To the best of our knowledge, this is the first integrated study among the Russian population aimed at revealing the original predictors of COVID-19-related lung damage severity.

## 2. Results

**Figure 1:**
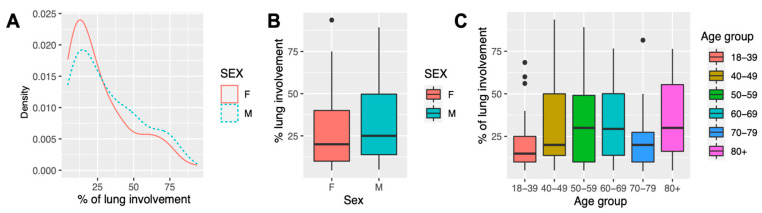
COVID-19-related lung damage severity in cohort study. (**A**) Density plot of the percentage of lung involvement in the study cohort in male (M, blue dashed line) and female (F, red solid line) groups. (**B**) Box plot of the percentage of lung involvement in the study cohort in male (M, blue) and female (F, red) groups, *p* = 0.193, *t*-test. (**C**) Box plot of the percentage of lung involvement in the study cohort among different age groups, *p* = 0.229, ANOVA. Black dots represent outliers.

A total of 151 patients, aged 24–96, with confirmed SARS-CoV-2 infection were included in the study cohort (Table 1). The median age was 57 [46.5–69] years; 73 patients (48%) were women. The percentage of lung involvement was characterized by CT scans and was 29.9 ± 1.83 (20 [10–43]) % in the studied cohort, with no significant differences between sexes (*p* = 0.193, *t*-test) or age groups (*p* = 0.229, analysis of variance (ANOVA)) (Figure 1).

To identify the genetic prognostic determinants of COVID-19-related lung involvement severity, patients were categorized according to the percentage of lung involvement on CT scans into “mild” (≤25% of lung involvement) and “moderate to severe” groups (>25% of lung involvement, hereinafter referred to as “severe”) (Table 1). Again, no significant differences in age and sex distribution were found between “mild” and “severe” groups (*p* = 0.053 and *p* = 0.3258, Fisher’s Exact Test). Patients were genotyped for *ACE* (I/D), *NOS3* (rs2070744, rs1799983), *SERPINE1* (rs1799768)*, PLAU* (rs2227564) and *PLAUR* (rs344781, rs2302524) polymorphisms (Table 2).

Genotype and allele distribution of *ACE*, *NOS3*, *SERPINE1*, *PLAU* and *PLAUR* gene polymorphisms are presented in Table 3 and Table 4, respectively. Among the tested SNPs, only *PLAUR* SNP rs2302524 was significantly associated with the lung involvement severity: rs2302524 T/C genotype showed a protective effect, being enriched in the group of “mild” COVID-19 patients: OR 0.349 [0.147–0.773] (*p* = 0.0130), assuming a codominant model of inheritance. This tendency was sustained when patients were adjusted by sex and age using logistic regression: OR 0.315 [0.130–0.708] (*p* = 0.0069) in a codominant model of inheritance (T/C vs. T/T; the Akaike information criteria (AIC) 204.6); OR 0.345 [0.152–0.741] (*p* = 0.0081) in a dominant model of inheritance (T/C-C/C vs. T/T; AIC 203.1); or OR 0.321 [0.133–0.719] (*p* = 0.0077) in the overdominant model of inheritance (T/C vs. T/T-C/C; AIC 202.9). None of the estimated *NOS3* or *PLAUR* haplotypes was significantly associated with lung damage severity groups (Table S1).

Among the tested clinical routine blood test factors, a higher lung damage severity was significantly associated with higher activated partial thromboplastin time (APTT, *p* = 0.00105, *t*-test), CRP (*p* < 0.0001, *t*-test), D-dimer (*p* = 0.00114, *t*-test), fibrinogen levels (*p* < 0.0001, *t*-test) and increased thrombin time (*p* = 0.00993, *t*-test) (Table 1). The international normalized ratio (INR) and prothrombin time yielded no significant differences between “mild” and “severe” groups (Table 1). Since *PLAUR* rs2302524 was identified as the SNP, significantly associated with the lung damage severity, the serum level of uPAR protein was also tested, and it was significantly higher in the “severe” COVID-19 group (*p* < 0.0001, *t*-test, Table 1), indicating that uPAR may serve as a good indicator of the disease severity. Of note, no significant association between uPAR serum level and tested *PLAUR* SNPs (rs344781 and rs2302524) or other tested SNPs was found (Figure S1, Table S2). suPAR positively correlated with CRP level (R = 0.45, Figure S2).

**Figure 2:**
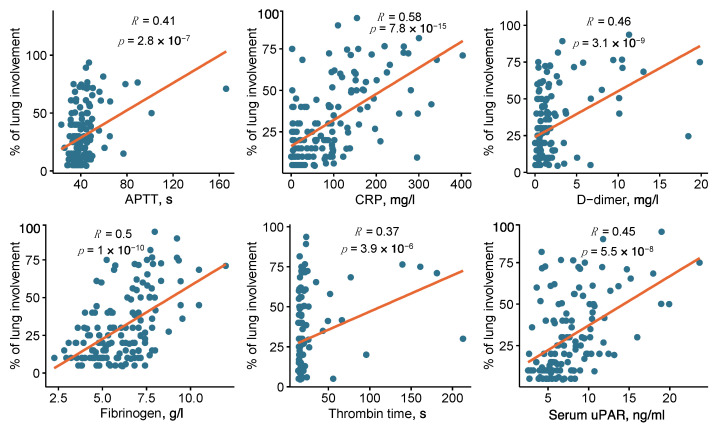
Correlation plots for the blood test parameters and lung damage severity (% of lung involvement). The Spearman correlation coefficients (R) and the corresponding *p*-values (*p*) are shown. The red line represents the linear regression fit, and the grey area represents the 95% confidence intervals. APTT, activated partial thromboplastin time; CRP, C-reactive protein.

**Figure 3:**
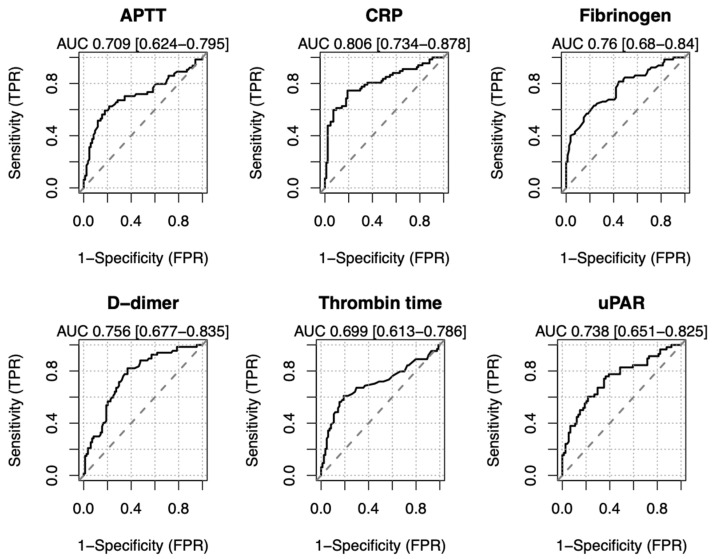
Empirical receiver operating characteristic (ROC) curves of the blood test parameters in predicting lung damage > 25%. The areas under the ROC curves (AUC) [95% confidence intervals] are provided for each parameter. The dashed line represents the chance line. APTT, activated partial thromboplastin time; CRP, C-reactive protein; FPR, the false positive rate; TPR, the true positive rate.

Additionally, we tested for a correlation between the significant blood test parameters and the percentage of lung damage severity. All the tested parameters demonstrated a significant positive correlation with lung damage severity, with the strength of correlation that decreased in the following order: CRP (R = 0.58), fibrinogen (R = 0.50), D-dimer (R = 0.46), uPAR (R = 0.45), APTT (R = 0.41), thrombin time (R = 0.37) (Figure 2). To evaluate the diagnostic performance of the studied parameters in predicting the lung damage > 25%, receiver operating characteristic (ROC) curves were plotted, and the areas under the ROC curves (AUC) were analyzed (Figure 3). The best predictive capability was observed for CRP (AUC 0.806 [0.734–0.878]); fibrinogen, D-dimer and uPAR serum levels showed comparable performances (AUC 0.76 [0.68–0.84], AUC 0.756 [0.677–0.835], AUC 0.738 [0.651–0.825]) in predicting COVID-19-related lung damage severity. The AUC for APTT and thrombin time were 0.709 [0.624–0.795] and 0.699 [0.613–0.786], respectively. An earlier proposed 6 ng/mL cut-off value for uPAR serum level in clinics [38,39] resulted in 77% sensitivity and 55% specificity of predicting the COVID-19-induced lung damage > 25% (Figure S3).

**Figure 4:**
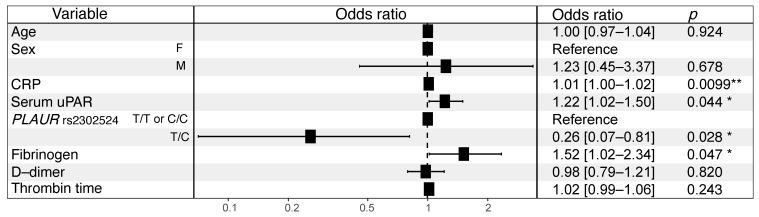
Forest plot of multivariable logistic regression model for factors associated with COVID-19-related lung damage severity. The horizontal lines correspond to odds ratio [95% confidence intervals] of “severe” vs. “mild” lung damage for each variable included in the association study. CRP, C-reactive protein. * *p* < 0.05; ** *p* < 0.01.

Finally, to identify independent prognostic factors of COVID-19-related lung damage severity, a multivariable logistic regression was performed, with age, sex, CRP, fibrinogen, D-dimer, thrombin time, uPAR serum level and *PLAUR* rs2302524 as independent variables. Among the studied parameters, CRP (OR 1.012 [1.003–1.022], *p* = 0.00992), serum uPAR (OR 1.219 [1.016–1.498], *p* = 0.0444) and fibrinogen (OR 1.517 [1.018–2.341], *p* = 0.0477) levels were found to be independent predictors of lung damage severity, while the T/C genotype of *PLAUR* rs2302524 was an independent predictor of a less severe lung damage (OR 0.258 [0.071–0.811], *p* = 0.0276) (Figure 4).

## 3. Discussion

Uncovering the risk factors for COVID-19 disease severity may enable early patient stratification and timely treatment implementation and thereby may improve the patient outcome and reduce the COVID-19-related healthcare burden. To address this question, we carried out a cohort study on 151 SARS-CoV-2-infected symptomatic adult patients (Figure 1), who were categorized according to the percentage of lung involvement on CT scans into “mild” (≤25% of lung involvement) and “severe” groups (>25% of lung involvement) and genotyped for candidate polymorphisms of *ACE* (I/D), *NOS3*, *SERPINE1, PLAU* and *PLAUR* genes. We identified *PLAUR* rs2302524 as the SNP associated with lung involvement in COVID-19 patients. The T/C genotype of *PLAUR* rs2302524 was independently associated with less severe lung damage (OR 0.258 [0.071–0.811]). Alongside elevated routine blood test parameters (CRP, fibrinogen), soluble uPAR serum levels were independently associated with more severe lung damage in COVID-19 patients (Figure 4). Here, we present the first integrated study among the Russian population aiming to reveal the genuine predictors of COVID-19-related lung damage severity.

Previous research identified several COVID-19 risk variables, including coagulation disorders, in patients with severe COVID-19 [17]. The routinely analyzed coagulation parameters in clinics comprise platelet count, fibrinogen, D-dimer, thrombin time, APTT, prothrombin time and INR [17]. High fibrinogen and D-dimer (fibrin degradation product) blood levels in COVID-19 patients are well-established mortality prognostic factors [7,40]. Fibrinogen and D-dimer levels in severe COVID-19 patients generally tend to be higher than in non-severe COVID-19 patients; the same tendency is observed in patients admitted to intensive care units (ICU) compared to non-ICU patients [41,42,43,44]. Longer prothrombin time and APTT has been also associated with COVID-19 mortality [45], although they remain quite controversial in determining COVID-19 severity, since they were found to be elevated [44] or unchanged [42,43,46] in severe COVID-19 patients compared to non-severe ones. Our data are in line with the recently published results [7,40,41,42,43,44,45,46]: we found that APTT, thrombin time, fibrinogen and D-dimer levels, but not INR or prothrombin time, are significantly higher in “severe” patients with more than 25% lung involvement as compared to “mild” COVID-19 patients (Table 1). Here we demonstrate that D-dimer and fibrinogen exhibit a moderate positive correlation with the percentage of lung involvement on CT in hospitalized COVID-19 patients (Figure 2). Interestingly, fibrinogen level revealed a stronger, albeit still moderate, correlation with the percentage of lung involvement (Figure 2) and a better lung damage predictive capability (Figure 3) compared to the other coagulation parameters tested; in multivariable analysis, fibrinogen was independently associated with COVID-19-related lung damage severity (OR 1.517 [1.018-2.341]) (Figure 4). These findings highlight the potential diagnostic importance of fibrinogen in predicting and monitoring COVID-19-induced lung damage.

Upon infection, SARS-CoV-2 induces a prominent inflammatory response largely correlating with the disease severity, with devastating consequences, such as hyperinflammation, cytokine storm, tissue damage and multi-organ failure [47]. CRP is an acute phase protein, routinely utilized as a biomarker of infection, inflammatory processes and cardiovascular events [48]. Monitoring the CRP blood level provides a valuable predictive opportunity in prognosing COVID-19-related mortality and severity [49,50]. Our data indicate that the mean CRP level is almost three times higher in the “severe” group relative to the “mild” COVID-19 group (Table 1). CRP blood level positively correlates with the percentage of lung involvement in COVID-19 patients on CT scans (Figure 2) and could be used for predicting lung damage >25% (Figure 3); in multivariable analysis, CRP remains independently associated with COVID-19-related lung damage severity (OR 1.012 [1.003–1.022]) (Figure 4).

There is a growing body of evidence suggesting that particular genotypes might account for predisposition to a more severe COVID-19 clinical course [51,52,53,54]. The overarching premise of such studies is that the severity of COVID-19 is (at least partially) genetically determined in each infected individual and that inter-individual genetic variability can explain various responses to viral infection in a population. In our screening study, *PLAUR* rs2302524 was found to be independently associated with the severity of lung involvement in COVID-19 patients (Table 3, Figure 4). *PLAUR* encodes urokinase receptor uPAR, and rs2302524 SNP translates into a uPAR missense variant with Lys220Arg substitution in the protein’s third domain (DIII); DIII of uPAR is engaged in uPA binding [55]. By interacting with uPA, uPAR plays a crucial role in producing serine protease plasmin [23,56]. uPAR has been linked to a variety of physiological processes, including cell differentiation, proliferation, migration and fibrinolysis [23,56]. It may also be involved in the pathogenesis of airway remodeling, lung injury and pulmonary fibrosis [19,57,58], which raises the possibility that uPAR is an important player in respiratory diseases [59,60,61]. *PLAUR* rs2302524 was previously linked to asthma susceptibility and to a decline in lung function in asthma [62], basal epithelial proliferation in asthmatic individuals [63] and baseline lung functioning in smokers [64]. Meanwhile, the functional significance of Lys220Arg substitution in uPAR remains to be resolved.

Upon inflammatory stimuli, uPAR is cleaved by uPA or other extracellular proteases, and its soluble form is released in circulation [65,66]. Here we demonstrate that serum uPAR level positively correlates with the percentage of lung involvement in COVID-19 patients (Figure 2), serving to predict lung damage >25% (Figure 3), and is independently associated with COVID-19-related lung damage severity (OR 1.219 [1.016–1.498]) (Figure 4). These results are in agreement with the previously published studies demonstrating that soluble uPAR serum levels may serve as an early predictor of clinical severity and outcome in patients with COVID-19 [67,68,69,70,71]. Soluble uPAR testing in serum, which is now commercially available and reasonably priced, has been continuously integrated into clinical practice. suPAR screening was recommended to predict the risk for unfavorable outcomes in patients admitted to the emergency department to improve the early diagnosis and management of sepsis [38]. In a phase 3 COVID-19 clinical trial, high suPAR serum level (cut-off value of ≥6 ng/mL) was used to identify patients at risk of progressing to respiratory failure [72]. Our results agree well with these previously published data and ascertain that an earlier proposed 6 ng/mL cut-off value for uPAR serum level provides 77% sensitivity and 55% specificity for predicting the COVID-19-induced lung damage > 25% (Figure S3). These data demonstrate a diagnostic value of serum uPAR level in prognostication of lung damage in COVID-19 patients and their stratification.

Genome-wide association study in Icelanders coupled with protein serum analysis suggested that *PLAUR* rs2302524 could affect the uPAR serum level [73], although later these results were reevaluated, since aminoacid substitution can potentially lead to artifactual associations of particular genotypes with protein levels identified by SOMAscan assays due to the interference with aptamer binding (so-called epitope-binding artifacts) [74]. It was further shown that while *PLAUR* rs2302524 was significantly associated with uPAR serum level measured by SOMAscan assays, this variant was no longer significant in enzyme-linked immunosorbent assay (ELISA)-based analysis [74]. Our results corroborate the conclusion that *PLAUR* rs2302524 is not associated with serum uPAR level (Figure S1, Table S2). We suggest that *PLAUR* rs2302524 rather affects uPAR functioning in lung tissue, contributing to lung damage severity, but not to its expression or secretion level, while soluble uPAR increase in blood serum is a direct indicator of immune activation that strongly and independently correlates with the disease severity.

Our study has some limitations. First, the results might be limited by the sample size. Second, not all laboratory tests were done in all the patients; however, the data were missing completely at random, and no information about the underlying comorbidities in the study cohort was available. Third, the cohort might not be fully representative, as the study was a single center one. However, we believe that our analysis reveals several key trends that should be further taken into account in evaluating the severity of COVID-19-induced lung injury. This association retrospective cohort study is the first in Russia to examine the relationship between specific SNPs, uPAR serum level, commonly used blood test parameters, and the percentage of lung involvement on CT scans in COVID-19 patients. The obtained results could serve as justification for testing these new parameters as promising indicators of COVID-19 severity and might help identify the prospective therapeutic targets to improve patient outcome.

## 4. Materials and Methods

### 4.1. Study Design and Clinical Workflow

The present study was a single-center retrospective study carried out on a cohort of 151 symptomatic adult patients with confirmed SARS-CoV-2 infection by a polymerase chain reaction (PCR) test. All patients were admitted to the Medical Research and Educational Center of Lomonosov Moscow State University, Moscow, Russia, from June to August 2020.

The study was approved by the ethics committee of the Medical Scientific and Educational Center, Lomonosov Moscow State University (protocol # 7/20, issued on 20.05.2020). The patients signed informed consent to participate in the study. The local ethical committee approved the retrospective study of the collected material.

Demographic, clinical and laboratory data were recorded at admission. Nasopharyngeal swabs were collected from all patients, followed by reverse transcription PCR (RT-PCR) assay to confirm the SARS-CoV-2 infection. For RT-PCR, RNA was extracted using a PREP-NA extraction kit (product number P-002/1, DNA-Technology, Moscow, Russia) following manufacturer’s instructions, and SARS-CoV-2 was identified using the SARS-CoV-2/SARS-CoV Multiplex real-time PCR Detection Kit with positive and negative control samples (product number R3-P436-23/9, DNA-Technology, Moscow, Russia) following the manufacturer’s instructions. Routine blood tests were carried out for all patients, and the following parameters were evaluated: CRP, prothrombin time, Quick prothrombin time, thrombin time, fibrinogen, D-dimer levels, APTT and INR.

### 4.2. Chest CT Protocol and Quantitative Assessment of Lung Involvement

A CT scan of lungs and chest organs was performed on a 32-slice Somatom Scope CT scanner (Siemens, Munich, Germany) with a slice thickness of 1 mm. A standard CT protocol was used with the following scanning parameters: 120 kV tube voltage, automatic modulation of the tube current in the range of 200–400 mA; for repeated CT scans, a low-dose CT protocol was used with the following scanning parameters: 100 or 110 kV tube voltage settings, automatic modulation of the tube current in the range of 40–120 mA. The standard CT protocol resulted in the average radiation exposure of 3.9 ± 0.4 mSv, the low-dose CT protocol delivered 0.9 ± 0.2 mSv average radiation exposure. CT examinations were performed upon the patient admission and hospital discharge; during the admission period they were repeated when clinically necessary, but at least once every 5 days.

Syngo.via workstations (Siemens, Munich, Germany) were used for CT processing and analysis. When processing and describing CT data, a semi-quantitative scale for assessing the infiltration and consolidation volume and zones was used, recommended by the Interim Guidelines of the Ministry of Health of the Russian Federation, adapted from the International Protocols and enriched with local experience [75]. For an accurate quantitative analysis of the COVID-19-related lung parenchyma lesions, the “Gamma Multivox” software package (Multivox, Moscow, Russia) was used. The software performed automatic color coding and counted the volumes of ground-glass opacities and consolidation zones on CT images, analyzing their percentage relative to the total lung volume for each patient; the sum of the volume percentages of ground-glass opacities and consolidation zones relative to the total lung volume was referred to as the percentage of lung involvement. The maximum value of all available measurements was used for subsequent analysis.

### 4.3. DNA Extraction and Genotyping

Genomic DNA was extracted from EDTA-stabilized peripheral venous blood using the PREP-GS Genetics DNA Extraction Kit (Product number P-023/4EU, DNA-Technology, Moscow, Russia) following the manufacturer’s instructions. *ACE* (I/D), *NOS3* (rs2070744, rs1799983), *SERPINE1* (rs1799768), *PLAU* (rs2227564) and *PLAUR* (rs344781, rs2302524) polymorphism (Table 2) genotyping was performed using standard kits with allele-specific primers (DNA-Technology, Moscow, Russia) following the manufacturer’s instructions. Quantitative PCR was carried out on a DT-96 real-time PCR device (DNA-Technology, Moscow, Russia). The thermal cycling program was as follows: a 2 min denaturating step at 80 °C, a 5 min denaturating step at 94 °C, followed by 5 amplification cycles consisting of 30 s denaturating at 94 °C, 15 s of annealing and elongation at 67 °C, followed by 40 amplification cycles consisting of 5 s denaturating at 94°C, 15 s of annealing and elongation at 67 °C. Genotypes were determined by the melting temperatures of amplification products in FAM and HEX channels. Minor allele frequency in the population was imported from the Genome Aggregation Database (gnomAD, version 3.1.2, https://gnomad.broadinstitute.org/ (accessed on 12 September 2022)) and [37].

### 4.4. uPAR ELISA

Levels of soluble uPAR in serum samples from the studied cohort were measured by ELISA (Human uPAR ELISA Kit, ab246549, Abcam, Cambridge, UK), following the recommended protocol.

### 4.5. Statistical Analysis

The data were analyzed with R (version 4.2.1) with the epitools (version 0.5-10.1), genetics (version 1.3.8.1.3), haplo.stats (version 1.8.9), ROCit (version 2.1.1), forestmodel (version 0.6.2), ggpubr (version 0.4.0) and ggplot2 (version 3.3.6) packages in R Studio environment (2022.07.1, PBC, Boston, MA, USA). Categorical variables (age, sex, genotype frequency) were described as numbers and percentages and compared using the Fisher’s Exact Test. Patients were grouped by age as: younger than 40 years, 40–49 years, 50–59 years, 60–69 years, 70–79 years, and 80 years or older. Continuous variables were reported as mean ± SEM or median [interquartile range] and compared by *t*-test (two groups) or one-way ANOVA (more than two groups). Hardy-Weinberg equilibrium (HWE) was assessed for all SNPs by the Fisher’s Exact Test using HWE.exact() function of the genetics package, and the hypothesis for the HWE in this population was retained for all SNPs (*p*-values > 0.05). The *p* values for the HWE are shown in Table 2.

A measurement of disease severity was tested for association with SNPs: patients were categorized according to the percentage of lung involvement on CT scans into “mild” (≤25% of lung involvement) and “moderate to severe” groups (>25% of lung involvement, referred to as “severe”). Genotype and allele frequencies between “mild” and “severe” groups were compared for significance using Fisher’s Exact Test using the fisher.test() function. OR with 95% confidence intervals were used to describe the strength of association based on the codominant inheritance model (oddsratio() function); they were compared for significance using Fisher’s Exact Test and presented as OR [95% confidence interval]. For adjusting age and sex parameters, logistic regression was performed using the glm() function with co-dominant, dominant, recessive, over-dominant and additive models; the models that fit well the data (the lowest Akaike information criteria) are provided. An expectation maximization (EM) algorithm was applied to estimate haplotype frequency and haplotype–trait association using the haplo.em() and haplo.glm() functions of the haplo.stats package.

For the correlation analysis, Spearman’s rank correlation between the percentage of lung involvement severity and the predictors (APTT, CRP, D-dimer, fibrinogen, thrombin time, uPAR) was performed. Predictive performance of continuous parameters (APTT, CRP, D-dimer, fibrinogen, thrombin time, uPAR) in predicting the severity of lung involvement > 25% was assessed using the area under the receiver operating characteristic (ROC) curve (AUC) via rocit() function of ROCit package with empirical method. To identify the independent prognostic factors of COVID-19-related lung involvement severity, a multivariable logistic regression was performed using the glm() function, with age, sex, CRP, Fibrinogen, D-dimer, thrombin time and *PLAUR* rs2302524 as independent variables and lung involvement severity (≤25% or >25%) as the binary dependent variable; the results were plotted using forest_model() function of forestmodel package. The level of significance was set at *p* < 0.05.

## Figures and Tables

**Table 1:** Demographic, clinical and laboratory characteristics of patients enrolled in the study. Data are presented as n (%), median [interquartile range] or mean ± SEM. *p*-values for comparisons between “mild” and “severe” groups are provided. APTT, activated partial thromboplastin time; CRP, C-reactive protein; INR, international normalized ratio. ** *p* < 0.01; **** *p* < 0.0001.

Variable	Total (*n* = 151)	“Mild” COVID-19 (*n* = 84)	“Severe” COVID-19 (*n* = 67)	*p*-Value
Age	57 [46.5–69]	56 [43.8–69.2]	58 [51–67.5]	0.053
<40	22 (15%)	17	5	
40–49	25 (17%)	15	10	
50–59	40 (26%)	17	23	
60–69	29 (19%)	14	15	
70–79	21 (14%)	15	6	
80+	14 (9%)	6	8	
Female	73 (48%)	44 (52%)	29 (43%)	0.3258
Male	78 (52%)	40 (48%)	38 (57%)	
% of lung involvement	29.9 ± 1.829	13.3 ± 0.681	50.8 ± 2.130	7.61 × 10^−28^ ****
APTT, s	41.8 ± 1.212	37.9 ± 0.744	46.8 ± 2.502	0.00105 **
CRP, mg/L	86.3 ± 6.95	46.6 ± 5.56	136.1 ± 11.45	2.91 × 10^−10^ ****
D-dimer, mg/L	1.88 ± 0.255	1.10 ± 0.240	2.85 ± 0.465	0.00114 **
Fibrinogen, g/L	6.06 ± 0.142	5.36 ± 0.149	6.94 ± 0.217	2.20 × 10^−8^ ****
INR	1.23 ± 0.049	1.15 ± 0.026	1.33 ± 0.104	0.102
Prothrombin time, s	17.2 ± 0.754	16.0 ± 0.392	18.6 ± 1.597	0.115
Quick prothrombin time, %	89.6 ± 1.35	87.9 ± 1.79	91.6 ± 2.04	0.179
Thrombin time, s	23.3 ± 2.28	17.5 ± 1.08	30.9 ± 4.95	0.00993 **
uPAR serum level, ng/mL	7.61 ± 0.328	6.20 ± 0.277	9.40 ± 0.580	3.43 × 10^−6^ ****

**Table 2:** SNPs included in the study. HWE was assessed for all SNPs by the Fisher’s Exact Test. Minor allele frequency in the population was imported from the Genome Aggregation Database. HWE, Hardy-Weinberg equilibrium; SNPs, single nucleotide polymorphisms.

Gene (RefSeq Accession Number)	rs	Variant	Type	Chromosome	Minor Allele Frequency	*p*-Values for HWE
*ACE* (NG_011648.1)	rs4646994	287bp Ins>Del	Intron variant	17	0.435 ^†^	0.516
*NOS3* (NG_011992.1)	rs2070744	c.-786T>C	Promoter variant	7	0.2998	0.611
*NOS3* (NG_011992.1)	rs1799983	c.894G>T; p.Glu298Asp	Missense variant	7	0.2446	0.705
*SERPINE1* (NG_013213.1)	rs1799768 ^‡^	c.-675 4G>5G; c.-1969_-1968insG	Promoter variant	7	0.370 ^†^	1
*PLAU* (NG_011904.1)	rs2227564	c.422C>T; p.Pro141Leu	Missense variant	10	0.1952	0.470
*PLAUR* (NG_032898.1)	rs344781	c.-516T>C	Promoter variant	19	0.2028	0.417
*PLAUR* (NG_032898.1)	rs2302524	g.43652320T>C; c.659A>G; p.Lys220Arg	Missense variant	19	0.1683	0.538

^†^ Allele frequencies from data reported in [37]; ^‡^ Variant also known as rs1799762, rs1799889 or rs34857375.

**Table 3:** Genotype distribution in “mild” and “severe” COVID-19 patients. *p*-values as compared by Fisher’s Exact Test are provided. CI, confidence interval; del, deletion; ins, insertion; OR, odds ratio; SNP, single nucleotide polymorphism. * *p* < 0.05.

SNP	Genotype	Genotype Frequencies, n (%)		Fisher’s Exact Test *p*-Value	OR [95% CI]	Fisher’s Exact Test *p*-Value
		“Mild” COVID-19	“Severe” COVID-19			
*ACE*, rs4646994	insins	19 (23%)	20 (30%)	0.3358	1	
	insdel	49 (58%)	31 (46%)		0.604 [0.276–1.315]	0.2377
	deldel	16 (19%)	16 (24%)		0.951 [0.368–2.454]	1.0000
*NOS3*, rs2070744	TT	30 (36%)	26 (39%)	0.6566	1	
	CT	41 (49%)	28 (42%)		0.790 [0.385–1.617]	0.5871
	CC	13 (15%)	13 (19%)		1.152 [0.447–2.972]	0.8151
*NOS3*, rs1799983	GG	43 (51%)	28 (42%)	0.3233	1	
	GT	36 (43%)	31 (46%)		1.319 [0.669–2.614]	0.4918
	TT	5 (6%)	8 (12%)		2.406 [0.713–8.930]	0.2220
*SERPINE1*, rs1799768	4G4G	19 (23%)	19 (28%)	0.6349	1	
	4G5G	45 (54%)	31 (46%)		0.692 [0.312–1.524]	0.4243
	5G5G	20 (24%)	17 (25%)		0.852 [0.339–2.131]	0.8185
*PLAU*, rs2227564	CC	49 (58%)	42 (63%)	0.1957	1	
	CT	34 (40%)	21 (31%)		0.723 [0.361–1.430]	0.3909
	TT	1 (1%)	4 (6%)		4.190 [0.554–117.6]	0.1906
*PLAUR*, rs344781	TT	45 (54%)	37 (55%)	0.6836	1	
	TC	33 (39%)	23 (34%)		0.850 [0.423–1.692]	0.7272
	CC	6 (7%)	7 (10%)		1.409 [0.422–4.846]	0.5676
*PLAUR*, rs2302524	TT	53 (63%)	55 (82%)	0.0199 *	1	
	TC	28 (33%)	10 (15%)		0.349 [0.147–0.773]	0.0130 *
	CC	3 (4%)	2 (3%)		0.660 [0.074–4.495]	0.6790

**Table 4:** Allele frequencies in “mild” and “severe” COVID-19 patients. *p*-values as compared by Fisher’s Exact Test are provided. CI, confidence interval; del, deletion; ins, insertion; OR, odds ratio; SNP, single nucleotide polymorphism. * *p* < 0.05.

SNP	Allele	Allele Frequencies, n (%)		OR [95% CI]	Fisher’s Exact Test *p*-Value
		“Mild” COVID-19	“Severe” COVID-19		
*ACE*, rs4646994	ins	87 (52%)	71 (53%)	1	
	del	81 (48%)	63 (47%)	0.953 [0.604–1.504]	0.908
*NOS3*, rs2070744	T	101 (60%)	80 (60%)	1	
	C	67 (40%)	54 (40%)	1.018 [0.639–1.619]	1
*NOS3*, rs1799983	G	122 (73%)	87 (65%)	1	
	T	46 (27%)	47 (35%)	1.431 [0.875–2.345]	0.168
*SERPINE1*, rs1799768	5G	85 (51%)	65 (49%)	1	
	4G	83 (49%)	69 (51%)	1.087 [0.689–1.715]	0.730
*PLAU*, rs2227564	C	132 (79%)	105 (78%)	1	
	T	36 (21%)	29 (22%)	1.013 [0.579–1.762]	1
*PLAUR*, rs344781	T	123 (73%)	97 (72%)	1	
	C	45 (27%)	37 (28%)	1.043 [0.623–1.738]	0.8969
*PLAUR*, rs2302524	T	134 (80%)	120 (90%)	1	
	C	34 (20%)	14 (10%)	0.464 [0.230–0.892]	0.0261 *

## Data Availability

The full data table with all the clinical, biochemical and genotyping anonymous results is available from the OSF database (doi:10.17605/OSF.IO/KR4MC, https://osf.io/kr4mc/?view_only=abb65d8339d04234b9803c1d1fa97257 (accessed on 17 December 2022)).

## Supplementary Materials

The supporting information can be downloaded at: https://www.mdpi.com/article/10.3390/ijms232416210/s1.
