# Supplementary material for: The Association of PLAUR Genotype and Soluble suPAR Serum Level with COVID-19-Related Lung Damage Severity
Source: Int J Mol Sci. 2022 Dec 19;23(24):16210. doi: 10.3390/ijms232416210 (PMC9785175; doi:10.3390/ijms232416210)
Supplement: Supplementary file 1 [file ijms-23-16210-s001.zip › ijms-2008493-supplementary.pdf]

Supplementary Materials

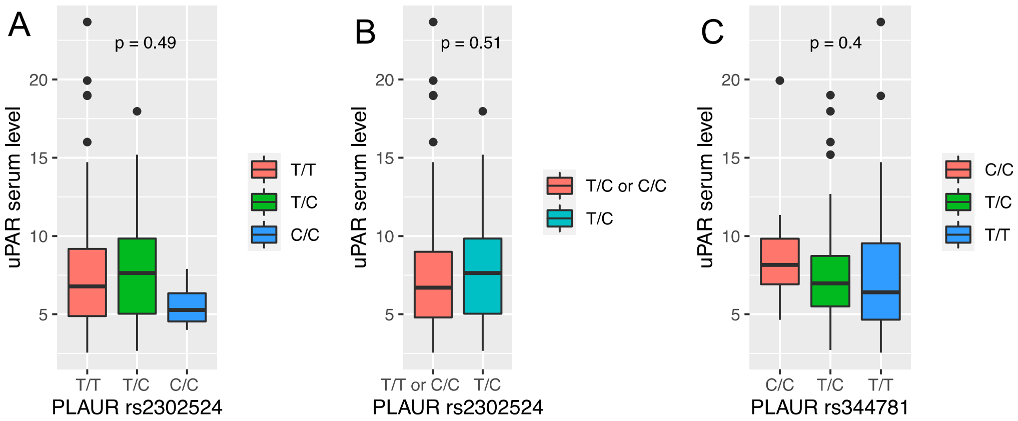

Figure S1. *PLAUR* SNP association with uPAR serum levels. (**A**) *PLAUR* rs2302524 association with uPAR serum levels in the codominant model of inheritance (T/T vs. T/C vs. C/C). (**B**) *PLAUR* rs2302524 association with uPAR serum level in the overdominant model of inheritance (T/C vs. T/T-C/C). (**C**) *PLAUR* rs344781 association with uPAR serum levels in the codominant model of inheritance (C/C vs. T/C vs. T/T). Data are presented as boxplots, black dots represent outliers, *p*-values as compared by ANOVA are provided.

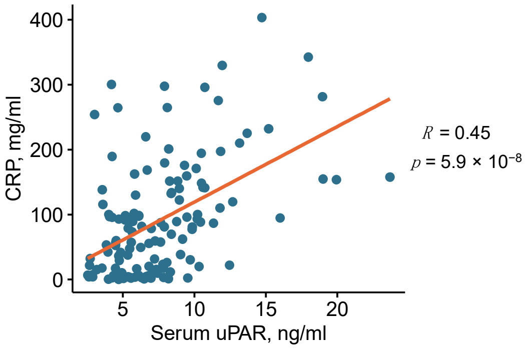

Figure S2. Correlation plot for the uPAR and CRP serum levels. The Spearman correlation coefficients (R) and the corresponding *p*-values (*p*) are shown. The red line represents the linear regression fit, and the grey area represents the 95% confidence intervals. CRP, C-reactive protein.

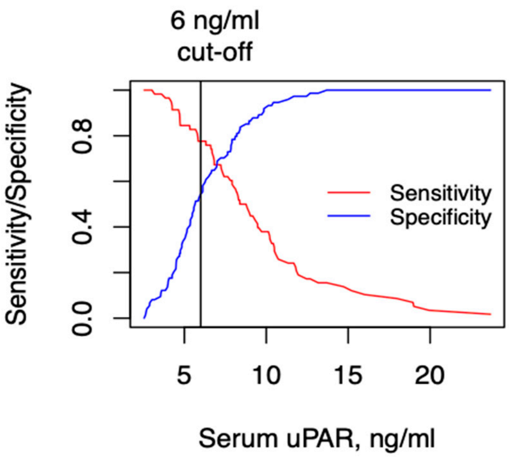

Figure S3. Sensitivity and specificity for predicting COVID-19-induced lung damage > 25% at different serum uPAR values. Previously proposed cut-off value of 6 ng/mL is shown.

Table S1. *PLAUR* and *NOS3* haplotype frequency estimation and association with lung damage severity (≤25% or >25%), adjusted by sex and age. The most prevalent haplotype was set as a reference. OR, odds ratio; CI, confidence intervals.

	***PLAUR*, rs344781**	***PLAUR*, rs2302524**	**Haplotype Frequency**	**OR [95% CI]**	***p*-value**
1	T	T	0.63679	1.00	
2	C	T	0.20427	1.31 [0.72–2.36]	0.3741
3	T	C	0.09168	0.54 [0.21–1.41]	0.2105
4	C	C	0.06726	0.39 [0.12–1.34]	0.1362
	***NOS3*, rs2070744**	***NOS3*, rs1799983**			
1	T	G	0.48564	1.00	
2	C	G	0.20641	1.37 [0.69–2.72]	0.3679
3	C	T	0.19425	1.19 [0.62–2.26]	0.6041
4	T	T	0.11369	2.56 [0.99–6.65]	0.0527

Table S2. Age- and sex-adjusted genotype association with uPAR serum levels with the assumption of a codominant model of inheritance. CI, confidence intervals; del, deletion; ins, insertion.

**SNP**	**Genotype**	**uPAR Serum Level, Mean ± SEM**	**Mean Difference [95% CI]**	***p*-value**
ACE, rs4646994	insins	7.73 ± 0.77	0	0.72
	insdel	7.82 ± 0.42	0.63 [−0.89–2.15]	
	deldel	7.09 ± 0.53	0.38 [−1.51–2.27]	
NOS3, rs2070744	TT	7.57 ± 0.5	0	0.28
	CT	7.26 ± 0.45	−0.31 [−1.70–1.08]	
	CC	8.72 ± 1.06	1.18 [−0.71–3.08]	
NOS3, rs1799983	GG	7.27 ± 0.49	0	0.77
	GT	7.99 ± 0.5	0.50 [−0.85–1.85]	
	TT	7.6 ± 0.94	0.15 [−2.25–2.55]	
SERPINE1, rs1799768	4G4G	7.94 ± 0.68	0	0.64
	4G5G	7.68 ± 0.49	−0.44 [−2.00–1.11]	
	5G5G	7.13 ± 0.57	−0.85 [−2.61–0.92]	
PLAU, rs2227564	CC	7.33 ± 0.39	0	0.29
	CT	8.2 ± 0.63	0.95 [−0.39–2.29]	
	TT	6.5 ± 0.89	−1.07 [−4.80–2.66]	
PLAUR, rs344781	TT	7.38 ± 0.45	0	0.42
	TC	7.64 ± 0.5	0.33 [−1.02–1.67]	
	CC	9.1 ± 1.36	1.65 [−0.83–4.13]	
PLAUR, rs2302524	TT	7.56 ± 0.39	0	0.44
	TC	7.98 ± 0.64	0.30 [−1.18–1.78]	
	CC	5.61 ± 0.85	−2.26 [−6.01–1.48]	
